# Predicted Impact of COVID-19 on Neglected Tropical Disease Programs and the Opportunity for Innovation

**DOI:** 10.1093/cid/ciaa933

**Published:** 2020-09-28

**Authors:** Jaspreet Toor, Emily R Adams, Maryam Aliee, Benjamin Amoah, Roy M Anderson, Diepreye Ayabina, Robin Bailey, Maria-Gloria Basáñez, David J Blok, Seth Blumberg, Anna Borlase, Rocio Caja Rivera, María Soledad Castaño, Nakul Chitnis, Luc E Coffeng, Ronald E Crump, Aatreyee Das, Christopher N Davis, Emma L Davis, Michael S Deiner, Peter J Diggle, Claudio Fronterre, Federica Giardina, Emanuele Giorgi, Matthew Graham, Jonathan I D Hamley, Ching-I Huang, Klodeta Kura, Thomas M Lietman, Tim C D Lucas, Veronica Malizia, Graham F Medley, Aronrag Meeyai, Edwin Michael, Travis C Porco, Joaquin M Prada, Kat S Rock, Epke A Le Rutte, Morgan E Smith, Simon E F Spencer, Wilma A Stolk, Panayiota Touloupou, Andreia Vasconcelos, Carolin Vegvari, Sake J de Vlas, Martin Walker, T Déirdre Hollingsworth

**Affiliations:** 1 Big Data Institute, Li Ka Shing Centre for Health Information and Discovery, Oxford, United Kingdom; 2 Department of Tropical Disease Biology, Liverpool School of Tropical Medicine, Liverpool, United Kingdom; 3 Mathematics Institute, University of Warwick, Coventry, United Kingdom; 4 Zeeman Institute for Systems Biology and Infectious Disease Epidemiology Research, University of Warwick, Coventry, United Kingdom; 5 Centre for Health Informatics, Computing and Statistics, Lancaster University, Lancaster, United Kingdom; 6 London Centre for Neglected Tropical Disease Research, Department of Infectious Disease Epidemiology, Imperial College London, London, United Kingdom; 7 Medical Research Council Centre for Global Infectious Disease Analysis, Department of Infectious Disease Epidemiology, School of Public Health, Imperial College London, London, United Kingdom; 8 The DeWorm3 Project, Natural History Museum, London, United Kingdom; 9 Faculty of Infectious and Tropical Diseases, London School of Hygiene and Tropical Medicine, London, United Kingdom; 10 Department of Public Health, Erasmus University Medical Center Rotterdam, Rotterdam, The Netherlands; 11 Francis I Proctor Foundation, University of California, San Francisco, California, United States of America; 12 Department of Biological Sciences, University of Notre Dame, Notre Dame, Indiana, United States of America; 13 Department of Epidemiology and Public Health, Swiss Tropical and Public Health Institute, Basel, Switzerland; 14 University of Basel, Basel, Switzerland; 15 The School of Life Sciences, University of Warwick, Coventry, United Kingdom; 16 Department of Ophthalmology, University of California, San Francisco, California, United States of America; 17 Centre for Mathematical Modelling of Infectious Disease, London School of Hygiene and Tropical Medicine, London, United Kingdom; 18 Department of Epidemiology & Biostatistics, University of California, San Francisco, California, United States of America; 19 School of Veterinary Medicine, Faculty of Health and Medical Sciences, University of Surrey, Guildford, United Kingdom; 20 Department of Statistics, University of Warwick, Coventry, United Kingdom; 21 London Centre for Neglected Tropical Disease Research, Department of Pathobiology and Population Sciences, Royal Veterinary College, University of London, Hatfield, Hertfordshire, United Kingdom

**Keywords:** neglected tropical diseases, coronavirus, modeling

## Abstract

Due to the COVID-19 pandemic, many key neglected tropical disease (NTD) activities have been postponed. This hindrance comes at a time when the NTDs are progressing towards their ambitious goals for 2030. Mathematical modelling on several NTDs, namely gambiense sleeping sickness, lymphatic filariasis, onchocerciasis, schistosomiasis, soil-transmitted helminthiases (STH), trachoma, and visceral leishmaniasis, shows that the impact of this disruption will vary across the diseases. Programs face a risk of resurgence, which will be fastest in high-transmission areas. Furthermore, of the mass drug administration diseases, schistosomiasis, STH, and trachoma are likely to encounter faster resurgence. The case-finding diseases (gambiense sleeping sickness and visceral leishmaniasis) are likely to have fewer cases being detected but may face an increasing underlying rate of new infections. However, once programs are able to resume, there are ways to mitigate the impact and accelerate progress towards the 2030 goals.

## Teaser

The COVID-19 pandemic has disrupted neglected tropical disease programs. Modelling shows the impact of this will vary across diseases. Once interventions are reintroduced, there are opportunities for mitigating the delay and accelerating progress towards the 2030 goals.

The coronavirus disease 2019 (COVID-19) pandemic will have wide-reaching implications for health systems and programs, including among populations in whom neglected tropical diseases (NTDs) are endemic. In the short term, preliminary World Health Organization (WHO) guidance advises that NTD surveys, active case detection activities, and mass drug administration (MDA) campaigns should be postponed, while support for prompt diagnosis, treatment, and essential vector control should continue where possible [1]. The impact of program disruptions on the hard-won gains of reduced infection, morbidity, and mortality, and therefore on elimination timelines, is uncertain. Mathematical modelling, such as that carried out by the NTD Modelling Consortium, can provide quantitative insights on how NTD programs could be impacted by the delays while showing potential catch-up strategies to mitigate impacts (ntdmodelling.org/covidinterruption.pdf). These models suggest that the impact on some NTDs can be mitigated in the years to come, provided the delay is minimal and that prompt remedial (and in some cases novel) action is taken.

During a disruption to interventions, the transmission of infectious diseases will gradually rise back towards the preintervention level unless interventions are reintroduced. The resulting rate of resurgence in prevalence depends on the rate at which new infections occur, which is driven by the natural histories of the disease and the local transmission conditions. The resurgence rates for many NTDs are relatively slow, particularly when compared with other infectious diseases, such as measles and malaria, meaning that the impact of a short disruption to NTD programs will accrue gradually. High-transmission areas face the greatest risk as resurgence will be faster in these areas. Additionally, the longer the delay of interventions, the greater the resurgence, and therefore the greater the rate of new infections. Once reintroduced, programs could consider intensifying the coverage or frequency of interventions, if politically and economically feasible, to mitigate the effects of the disruption.

**Figure 1.**
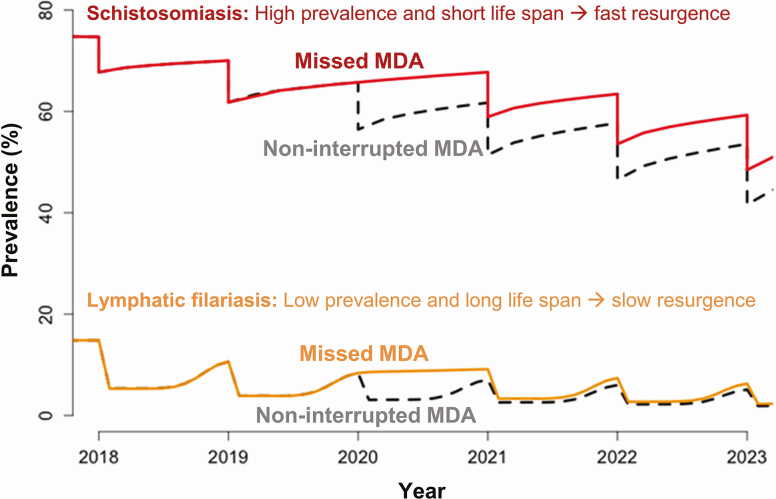
Modelling projections showing the impact of missing a round of MDA for schistosomiasis (*Schistosoma mansoni*; mean worm lifespan, 5.6 years) and (*Anopheles*-transmitted) lymphatic filariasis (*Wuchereria bancrofti*; mean worm lifespan, 8 years). Schistosomiasis starting at 75% baseline prevalence in school-aged children (5–14 years old) and treating 75% of school-aged children with praziquantel. Lymphatic filariasis starting at 15% baseline prevalence in the entire population and treating 65% communitywide with ivermectin and albendazole (assuming 30% long-lasting insecticidal nets coverage). Abbreviation: MDA, mass drug administration.

For the NTDs managed via MDA campaigns, the impact of delaying MDA will vary across diseases. For the helminth infections, the resurgence rate following a round of MDA is driven by the local transmission rate and the lifespan of the adult worm. Shorter-lived worms will have higher resurgence because resurgence rates are approximately proportional to 1/L, where L is the lifespan of the worm in the human host [2]. Hence, missing an annual MDA for soil-transmitted helminthiases (STH) and schistosomiasis programs (lifespans from 1 to 10 years [3–7]) will be more severe than for onchocerciasis and lymphatic filariasis (LF) (lifespans from 5 to 15 years [8, 9]) (Figure 1) (but unlike LF, onchocerciasis MDA treatments are not strongly adulticidal [10]). Similar analysis for trachoma estimated that the doubling time (time over which the number of cases double) for resurgence may vary from 1–2 months in highly endemic settings to 4–8 months in lower endemic settings [11], suggesting that trachoma programs face a greater resurgence risk relative to the helminths. The risk of trachoma resurgence will be greatest in high-prevalence settings, where a 1-year delay may extend elimination timelines by 2–3 years.

For NTDs for which intensified testing and case finding is the primary strategy, the resurgence rates are more difficult to estimate. This is due to uncertainties in the natural history of infection and, more importantly, because changes in the number of newly diagnosed cases are a consequence not only of new infections but also of how rapidly they are detected. Therefore, disruptions due to COVID-19 are likely to lead to fewer cases being detected, while the underlying rate of new infections increases. Where this happens, detections of outbreaks may be delayed, posing a challenge to health system responses. For visceral leishmaniasis (VL) in the Indian subcontinent, halting programs for 1 year likely causes incidence to revert to the rate occurring 1 year earlier, if the program is currently in the attack phase (ie, undertaking intense control measures). For settings that are already below the target incidence (<1 per 10 000 annually [12]) with less-intense measures, the setback could be up to 5 years, leading to an incidence above the elimination target in previously highly endemic settings. Local outbreaks may also be possible. For the gambiense form of human African trypanosomiasis (gHAT), incidence is likely to continue to decline if the disruption only lasts for 1 year but may increase in the second year of disruption, especially if diagnosis and treatment of cases presenting at health facilities are disrupted; modelling suggests transmission temporarily increased in Mandoul, Chad, during a 2-year screening break in 2007–2008 [13].

NTD programs are at different stages with both well-established and early programs being impacted by the disruption. Programs that have made good progress in reducing the levels of infection in a high-baseline setting may face a particular risk of faster resurgence, and so may lose the most. In contrast, more recently established programs may not have made such gains and so may lose less but will be closer to the baseline setting and at risk of returning to higher levels sooner. Further modelling of such specific settings is required to inform estimates of the impact.

Vector control offers the potential to mitigate the impact of a disruption to programs as it reduces the transmission rate of some infections, therefore slowing resurgence. However, the impact of vector control depends on the extent to which it is sustained during the disruption. While it is unlikely that new vector-control activities can be introduced during the COVID-19 pandemic, continuation of existing measures may help, as evident during program disruptions due to the West African Ebola outbreak in Guinea, where areas with vector control saw fewer gHAT cases after normal activities resumed [14]. For VL, the effect of suspending indoor residual spraying efforts should be expected to be more variable at local village levels, ranging from very limited impact to outbreaks as observed in Kosra, India [15, 16]. For LF, the use of bednets has a small impact relative to MDA and, in addition, bednets will not be effective in areas where the predominant vector species bites during the day [17, 18].

Interim guidance has recommended that water, sanitation, and hygiene (WASH) activities should continue during the postponement of mass interventions, with a greater focus on supporting implementation of COVID-19–related measures where needed, such as hand hygiene [1, 19]. The modelling of WASH on NTD transmission has received limited attention, but reviews based on current evidence from STH and trachoma programs show contrasting effects of WASH on infection risk, suggesting that it may have limited impact on reducing transmission [20–22].

When interventions are reintroduced, there may be an opportunity for NTD programs to accelerate progress towards the 2030 goals—for example, through biannual MDA for trachoma and onchocerciasis, or use of more effective drug combinations, such as the triple-drug regimen for LF in eligible settings [10, 11, 23]. Expansion of programs to communitywide treatment could be considered for STH and schistosomiasis in high-transmission settings [24, 25]. Increased active case detection could be implemented for VL and gHAT, alongside improved coverage of vector control (indoor residual spraying of insecticide for VL and tiny targets for gHAT [26, 27]). Such intensified strategies should be prioritized in high-transmission areas where they will be most beneficial, as well as in areas where programs may encounter longer delays.

The future course of the COVID-19 pandemic is uncertain, as is the public health response to its progression in different settings. Multiple disruptions to NTD programs are a possibility, and each disruption will have an impact on transmission. The duration and extent of these disruptions will affect their short- and long-term impact on achievement of the NTD goals and associated morbidity or mortality. Once NTD programs are able to resume, many financial and human resources will be constrained, hindering business-as-usual. However, targeting of catch-up strategies should be informed by up-to-date data, rather than implementing standard disease-specific interventions. Re-surveying areas once programs are resumed will also allow for empirical validation of model-based predictions. The future of NTD programs may necessitate novel approaches, such as integrated treatment, whereby multiple NTDs are targeted at once, and integrated surveys. Furthermore, there may also be opportunities to incorporate COVID-19 surveillance in NTD programs to aid in mitigating further COVID-19 outbreaks.

During the year in which NTD programs should be celebrating achievements in progress towards 2020 goals [28] and building towards even more ambitious goals for 2030 [29], COVID-19 will impact both the interventions and surveillance programs that have led to and revealed hard-won gains. Programs will want to resume as promptly as possible, but, as highlighted here, for some NTDs and in some settings business should not resume as normal. Rather, there is an opportunity to implement novel catch-up strategies in order to ensure programs remain on track. These efforts will require WHO leadership, flexible support from donors, and research to evaluate impacts of different approaches. In the context of limited resources for NTD programs and the multifactorial impact of COVID-19 on health systems, prioritization and rationalization of mitigation strategies can be used to respond to programmatic needs, strengthening NTD programs for the future.

## Notes

***NTD Modelling Consortium members*.** Emily R. Adams, Maryam Aliee, Benjamin Amoah, Roy M. Anderson, Diepreye Ayabina, Robin Bailey, Maria-Gloria Basáñez, David J. Blok, Seth Blumberg, Anna Borlase, Rocio Caja Rivera, María Soledad Castaño, Nakul Chitnis, Luc E. Coffeng, Ronald E. Crump, Aatreyee Das, Christopher N. Davis, Emma L. Davis, Sake J. de Vlas, Michael S. Deiner, Peter J. Diggle, Claudio Fronterre, Federica Giardina, Emanuele Giorgi, Matthew Graham, Jonathan I. D. Hamley, T. Déirdre Hollingsworth, Ching-I Huang, Klodeta Kura, Epke A. Le Rutte, Thomas M. Lietman, Tim C. D. Lucas, Veronica Malizia, Graham F. Medley, Aronrag Meeyai, Edwin Michael, Travis C. Porco, Joaquin M. Prada, Kat S. Rock, Morgan E. Smith, Simon E. F. Spencer, Wilma A. Stolk, Jaspreet Toor, Panayiota Touloupou, Andreia Vasconcelos, Carolin Vegvari and Martin Walker.

***Author contributions.*** This Viewpoints article arose from a virtual meeting for which all authors contributed analyses and discussions. T. D. H. and J. T. wrote the first draft. N. C., K. S. R., J. M. P., W. A. S., M. G. B., J. I. D. H., E. L. R., F. G., D. A., K. K., and P. J. D. edited the document in detail. All authors read and approved the final draft.

***Financial support.*** The NTD Modelling Consortium was supported by the Bill and Melinda Gates Foundation (grant number OPP1184344). S. B. acknowledges National Institutes of Health (NIH) grant R01 EY025350. L. E. C. acknowledges funding from the Dutch Research Council (NWO) (grant number 016.Veni.178.023). R. E. C., C. H., and K. S. R. acknowledge funding by the Bill and Melinda Gates Foundation through the Human African Trypanosomiasis Modelling and Economic Predictions for Policy (HAT MEPP) project (grant number OPP1177824). F. G. acknowledges funding from a European Marie Skłodowska-Curie fellowship (H2020-COFUND-2015-FP-707404). M. G. B. and J. I. D. H. acknowledge joint center funding for MRC GIDA (MRC Centre for Global Infectious Disease Analysis) (grant number MR/R015600/1) by the UK Medical Research Council (MRC) and the UK Department for International Development (DFID) under the MRC/DFID Concordat agreement, which is also part of the EDCTP2 program supported by the European Union. T. M. L. acknowledges funding from the NIH. E. M. acknowledges partial funding from the NIH (grant number RO1AI123245). T. C. receives salary from grants from the US NIH and from the Bill and Melinda Gates Foundation.

***Potential conflicts of interest.*** The authors: No reported conflicts of interest. All authors have submitted the ICMJE Form for Disclosure of Potential Conflicts of Interest.
